# Informing Women on Menopause and Hormone Therapy: *Know the Menopause* a Multidisciplinary Project Involving Local Healthcare System

**DOI:** 10.1371/journal.pone.0085121

**Published:** 2013-12-31

**Authors:** Serena Donati, Roberto Satolli, Cinzia Colombo, Sabrina Senatore, Rodolfo Cotichini, Roberto Da Cas, Stefania Spila Alegiani, Paola Mosconi

**Affiliations:** 1 Centro Nazionale di Epidemiologia, Sorveglianza e Promozione della Salute, Istituto Superiore di Sanità, Roma, Italia; 2 Agenzia Editoria Scientifica Zadig, Milano, Italia; 3 Laboratory for medical research and consumers involvement, IRCCS, Istituto di Ricerche Farmacologiche Mario Negri, Milano, Italia; University of Padova, Italy

## Abstract

**Background:**

Hormone therapy (HT) in the menopause is still a tricky question among healthcare providers, women and mass media. *Informing* *women* *about* *hormone* *replacement* *therapy* was a Consensus Conference (CC) organized in 2008: the project *Know* *the* *Menopause* has been launched to shift out the results to women and healthcare providers and to assess the impact of the cc’s statement.

**Methods:**

**And Findings**: The project, aimed at women aged 45-60 years, was developed in four Italian Regions: Lombardy, Tuscany, Lazio, Sicily, each with one Local Health Unit (LHU) as “intervention” and one as “control”. Activities performed were: survey on the press; training courses for health professionals; educational materials for target populations; survey aimed at women, general practitioners (GPs), and gynaecologists; data analysis on HT drugs’ prescription. Local activities were: training courses; public meetings; dissemination on mass media. About 3,700 health professionals were contacted and 1,800 participated in the project. About 146,500 printed leaflets on menopause were distributed to facilitate the dialogue among women and health care professionals. Training courses and educational cascade-process activities: participation ranged 25- 72% of GPs, 17-71% of gynaecologists, 14-78% of pharmacists, 34-85% of midwives. Survey: 1,281 women interviewed. More than 90% believed menopause was a normal phase in life. More than half did not receive information about menopause and therapies. HT prescription analysis: prevalence fell from 6% to 4% in five years. No differences in time trends before-after the intervention. Major limitations are: organizational difficulties met by LHU, too short time for some local activities.

**Conclusions:**

A huge amount of information was spread through health professionals and women. The issue of menopause was also used to discuss women’s wellbeing. This project offered an opportunity to launch a multidisciplinary, multimodal approach to menopause looking not only at pharmacological aspects, but also at quality of life and information.

## Introduction

Hormone therapy (HT) in the menopause is still a tricky question among health care providers, women and the mass media. During 2012 the North American Menopause Society [1] and the International Menopausal Society [2] updated their HT position Both evaluated pros an cons of the HT, and underlined that a careful assessment of individual risks and benefits should be discussed by each woman. 2013 opened with the publication of an editorial [3] about the United States Preventive Services Task Force (USPSTF) recommendations against using hormone therapy for the prevention of chronic conditions in postmenopausal women[4]. The editorial also commented on the Danish Osteoporosis Prevention Study DOPS [5] published after the systematic review [4], that showed cardiovascular benefits and no apparent harms; the methodological limits of the DOPS, underlined by the editorial’s authors, led them to conclude that the USPSTF recommendations remained sound. 

The systematic review updating the USPSTF recommendations [6] analysed the literature from January 2002 to November 2012 (nine trials published) to evaluate the effectiveness of HT in reducing risks for chronic conditions and adverse effects. Oestrogen plus progestin and oestrogen alone reduced the risk of fractures, but raised the risk for stroke, thromboembolic events, gallbladder disease, urinary incontinence. Oestrogen plus progestin increased the risk of breast cancer and probably dementia, while oestrogen alone reduced the risk of breast cancer. In the light of these findings, the USPSTF made a grade D recommendation against the use of combined oestrogen and progestin for the prevention of chronic conditions in postmenopausal women. 

Like in other industrialized countries, in Italy too the debate on HT is still lively, although the results of different studies over time have dampened the enthusiasm for drug treatments. Attention on the menopause seems to come to life when the results related to (new) drug treatments are published. This limits the quality and completeness of information for women focusing only on (new) drugs and not on the menopause as a normal phase of life, and often means women receive conflicting advice from different stakeholders. Although the importance of individual choice in decisions on HT is often emphasized - in terms of quality of life and risk assessment [7] - the debate on the type of information that women should receive remains weak.

Focusing on this issue, a Consensus Conference (CC) entitled “Informing women about hormone replacement therapy” was organized in Turin in May 2008 [8-10]. Agreement among members with different backgrounds - general practitioners, gynaecologists, epidemiologists, pharmacologists, public health experts, journalists, representatives of associations of citizens and patients - was formalized in a public final statement. The multidisciplinary nature of this process, the review of published scientific evidence, the analysis of women’s information needs and the evaluation of messages conveyed by the mass media supported the final statement. The key messages were: women need to be fully informed about the transient nature of menopausal symptoms, HT risks and benefits, and the availability of non-pharmacological interventions; HT is useful to treat menopausal symptoms, at the lowest dose for the shortest time; HT is not recommended to prevent chronic conditions of aging; the term "HRT" is misleading and "post-menopausal hormone therapy" should be the preferred definition [8]. Accordingly with the recent systematic review published by USPSTF [6] the CC results remain valid.

**Figure 1:**
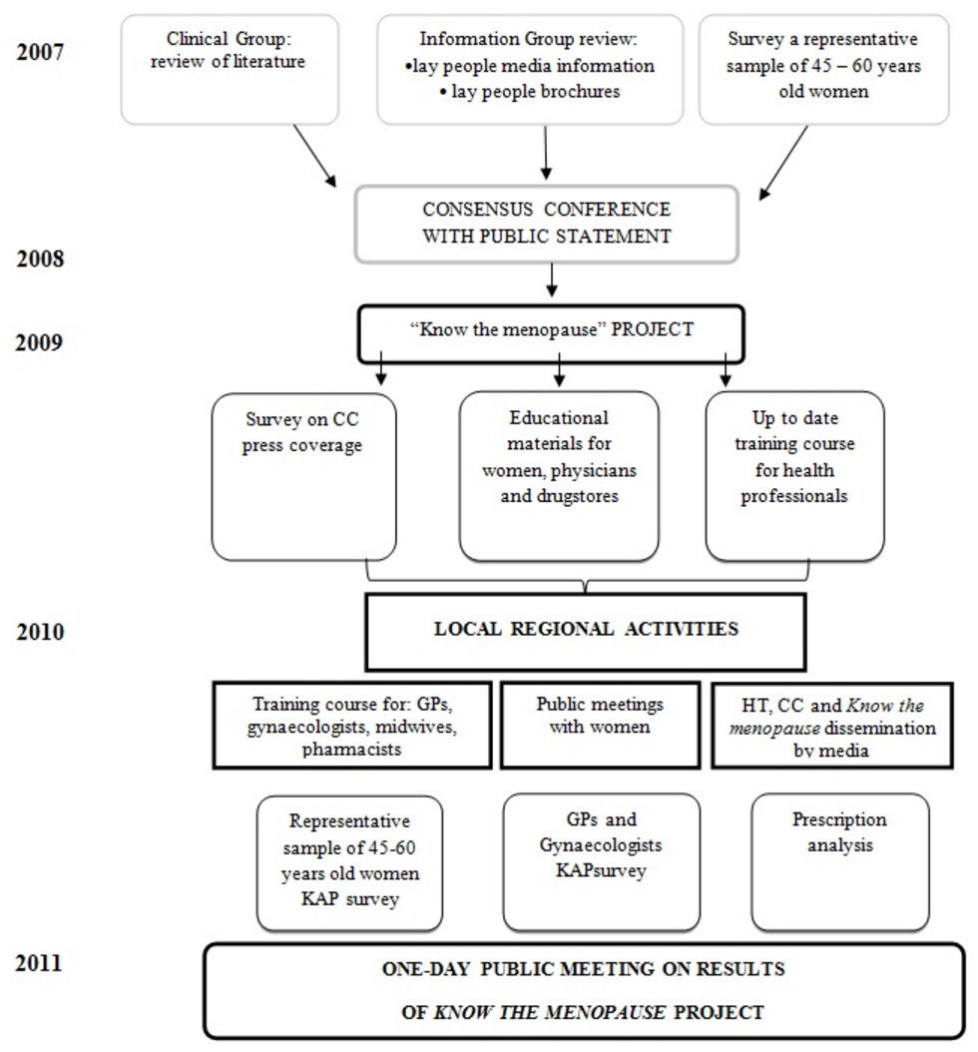
From the Consensus Conference to Know the menopause project. Legend: KAP=Knowledge, Attitude and Practice; HT=hormone therapy; CC=consensus conference; GP=general practitioner.

The results need to be shifted out to a larger context to reach the real world, where women and health care providers can discuss these issues in daily life. To assess the impact of the dissemination of the CC’s statement, a project known as *Conoscere la menopausa* (i.e. *Know the Menopause*) has been launched (Figure 1).

## Methods

### Ethics Statement

This project obtained the approval from the Ethics Committee of the Istituto Superiore di Sanità.

The project, aimed at women aged between 45 and 60 years, was launched in May 2009 and developed in four Italian Regions: Lombardy, Tuscany, Lazio, Sicily. Each region participated with one Local Health Unit (LHU) as “intervention unit”, in which the promoters endorsed different initiatives to implement the recommendations of the CC, and one LHU as “control unit”, with no implementation. It is a multi-modal, project that follows a cascade-process approach, developing activities involving National Health Service (NHS) staff and lay people, in particular women (Figure 1). The central activities, i.e. those organised and carried out directly by promoters, and local activities, organised and carried out directly by LHUs, are briefly described here. 

### Central activities

#### Survey on the press

Between the end of the CC and the start of the project *Know the menopause* the lay press was checked and we selected the articles published in a list of relevant lay press publications (health magazines, weekly news magazines, newspapers, women’s magazines) and some specialized journals for medical practitioners using the keywords ‘‘menopause’’, ‘‘hormone replacement therapy’’, and ‘‘hormones’’. All the journals had a a national coverage.

Two reviewers independently evaluated the articles, using a standardised form [11] considering headline, summary, pictures, length and the content of the article.

#### Training course

Residential and E-learning courses were offered to “intervention” LHUs under the accreditation system of Continuing Medical Education (CME). Modular educational activities were organized to provide health professionals (gynaecologists, midwives, general practitioners and pharmacists) with up-to-date information, and to promote appropriate communication skills. 

Scientific evidence on menopause and HT, and the CC recommendations were presented. A training section was entirely devoted to communication and counselling skills, with role-playing and discussion of cases. Each trainer received a *cascade training package* on CD for what they would teach once back in their LHUs.

An e-learning course was offered to physicians, midwives and pharmacists of the intervention LHUs. E-learning took place on the platform GOAL (Guidelines Online Assistant-Local), a software tool designed to do various activities, through completely online group work, to support the production, dissemination and implementation of the recommendations for clinical practice.

Finally, in each intervention LHU an interdisciplinary meeting was organized and participants evaluated the proactive dissemination of the CC’s recommendation, analysing strengths and limits, and identifying future developments.

#### Educational materials

A preliminary postal survey was done in participants LHUs to collect and evaluate the material already available. Educational material, defined on the basis of the CC’s recommendation, was then developed for two target populations of the LHU “intervention”: women aged 45-60 years and health professionals.

For the women, three focus groups (North, Central and South of Italy), moderated by a psychologist were formed to discuss knowledge and questions about menopause and HT and to evaluate the clarity, completeness and readability of a first draft of six-page leaflet. More technical material was developed and discussed with health professionals. Finally, a sticker was prepared with the logo of the project for the pharmacies.

#### Survey aimed at women

sample surveys targeting women aged 45-60 years, randomly selected from the electoral rolls, were done in intervention and control LHUs to investigate their knowledge, attitude and behavior about menopause and HT. Twenty trained interviewers administered structured questionnaires to all participants at their homes. At the beginning of the interview the interviewers, according with the protocol and the training received, read a short presentation of the aims of the study as reported on the front page of the questionnaire. Women provided the verbal informed consent to participate and the interviewers registered it on the record. According with the Ethics Committee this modality was considered the most appropriate and feasible.

The questionnaire was designed by a multidisciplinary team and pre-tested in the field. Menopausal history, HT and any other possible treatments, type and amount of information received, perceived quality and satisfaction, as well as personal habits/life style, health and socio-demographic characteristics were collected. Univariate and multivariate analysis were done centrally by project promoters, using STATA Package version 9.

#### Survey aimed at physicians

general practitioners and gynaecologists in intervention and control LHUs were surveyed to check their knowledge, attitude and behavior about menopause and HT. Health professionals were invited to participate and solicited by e-mail and mail. The questionnaire was sent by mail in Sicily, and administered through the GOAL platform in all other LHUs. 

#### Prescription analysis

the data on pharmaceutical use in Italy refer to HT drugs whose costs are covered by the NHS in the period 2000-2010. Each LHU collected and transmitted to the study group all HT prescriptions for women aged 45-60 years in the period 2006-2010 (about 465,000 aged 45-60 years women, 7% of the Italian population). Drugs are classified using the International Anatomical Therapeutic Chemical (ATC) classification. Drug consumption was expressed in defined daily doses (DDD), that is, the daily maintenance dose for adults for the drug’s main indication. The number of DDD was expressed as “DDD per 1,000 population per day” (i.e. the mean number of doses consumed daily by every 1,000 individuals). Time-series analysis was done to evaluate the effect of the intervention in the eight LHUs comparing prescription data before and after the intervention.

### Local LHU activities

Nine different type of actions were identified and shared with regional representatives to disseminate information throughout the territory of the intervention LHUs, promoting more knowledgeable health choices among women. Each intervention LHU had to implement at least four of the following type of action:

1. information and counselling during general practitioners’ consultations or family planning counselling;
2. information during cervical cancer screening;
3. counselling during gynaecology outpatient services;
4. information in pharmacies when people purchase medicines for menopause;
5. organization of thematic meetings at family planning clinics and other public places (communal spaces, pharmacies, schools, senior centres, etc.);
6. involvement of middle and high schools to promote educational courses according to Health Promoting Schools model;
7. involvement of local media (newspapers, local radio and TV) to promote articles in print, and/or meetings/discussions on menopause and the appropriate use of HT;
8. distribution of educational leaflets in LHU, offices of family physicians, pharmacies, meeting places, etc;
9. postal delivery of educational leaflets and information material on the project to the target women.

## Results

### The press survey

Over half of the evaluated articles suggested HT to cure menopause symptoms, 44% suggested it for prevention. Nearly two thirds (61%) proposed HT to prevent fractures, 53% cardiovascular diseases. Almost 60% mentioned HT’s risks, 40% did not. The most cited source of information was experts (65% of the articles), followed by clinical studies (41%) and congresses (35%). The information on HT is often unbalanced and incomplete, preventing women to make informed decisions.

### Participation in training courses

Participation in the residential training courses for health professionals ranged from 25% to 72% of GPs, 17-71% of gynaecologists, 14-78% of pharmacists, and 34-85% of midwives. Participation varied among intervention LHUs, depending also on the methods used to promote the meetings.

Participation in the E-learning courses also varied among LHUs, maybe because of the different ways of implementation: only two LHUs applied for CME credits for the e-learning course. In these two LHUs, the response rates were 16% and 58% of all the general practitioners, gynaecologists, midwives, nurses and pharmacists registered in the platform. Among the participants, 80% and 88% succeeded. The course with no CME credits had a very low participation rate.

### Women’s and health professionals’ surveys

A total of 1,281 women were interviewed. Almost 80% of the sample was married, and 48% had more than 8 years of education. Women were interviewed about their reproductive health history and 87% of them had at least one child. Fifty nine percent of the sample (749 women) reached menopause, 85% naturally and 14% as a consequence of surgery or radiant therapy. Menopausal status was defined as the absence of menstrual period for 12 consecutive months. Reported mean age at menopause is 49 years, while the median age is 50 years.

More than 90% of the respondents believed that menopause was a normal phase in a woman’s life and about 28% stated it was a good experience. Nevertheless, more than half the sample did not receive any information about menopause and possible therapies. Forty-eight percent of respondents reported having received information before the menopause, with higher percentages in intervention LHU (54%) compared to the control LHU (41%). In addition, the information in intervention LHU was offered by health professionals more often in a proactive way rather than following a specific request of the women (table 1). Table 1 describes some results regarding knowledge and attitude towards HT among women who reached menopause, in general women in the intervention LHUs have better knowledge on the subject compared to the women of the control LHUs.

**Table 1:** Results of survey among women.

	**LHU INTERVENTION**		**LHU CONTROL**		p-value*
	**N**	**%**	**N**	**%**	
**All sample: HT in menopause can cause adverse reactions**					
Agree	182	48	107	29	0.002
Not agree	26	7	41	11	0.552
Don’t know	155	41	203	55	0.006
Missing	19	5	16	4	0.932
**All sample: Even if there are a few problems, HT is a good opportunity for women**					
Agree	35	9	48	13	0.579
Not agree	240	63	133	36	0.000
Don’t know	89	23	170	46	0.000
Missing	18	5	16	4	0.961
**All sample: HT is a good way to prevent age-related symptoms**					
Agree	68	18	98	27	0.181
Not agree	159	42	67	18	0.001
Don’t know	136	36	186	51	0.007
Missing	19	5	16	4	0.932
**Women who received information: When did you receive information?**					
Before menopause	173	54	129	41	0.018
Menopause/post-menopause	138	43	185	58	0.008
Missing	7	2	3	1	0.892
**Women who received information: How did you receive information by health professionals?**					
Proactive offer	137	43	101	32	0.078
Following the woman’s request	106	33	143	45	0.061
No information from health professionals	66	21	69	22	0.886
Missing	9	3	4	1	0.863

As reported in the logistic regression model (table 2), women with high educational level, those who reached menopause, who reported menopausal symptoms and those who accessed Maternal and Child Health Services (MCHS) in the last five years, had a higher probability to be informed about menopause.

**Table 2:** Logistic regression model on the likelihood of receiving information about menopause.

**Interviewed women**	**Total**	**Women who received information about menopause**					
	**N 1.281**	**N 635**	**%**	**ORgr**	**(CI95%)**	**ORad**	**CI95%**
**Education**							
≤8 years	625	278	44	1		1	
≥8 years	656	357	54	1,49	1,20-1,86	1,63	1,29-2,07
**Menopause**							
Pre-menopause	532	214	40	1		1	
Menopause	370	213	58	2,02	1,54-2,64	1,41	1,00-1,99
Post-menopause	379	208	55	1,81	1,38-2,36	1,25	0,88-1,78
**Menopause symptoms**							
No	705	285	40	1		1	
Yes	576	350	61	2,28	1,82-2,86	1,98	1,45-2,68
**Gynaecologic check in the last 5 years**							
No	241	110	46	1			
Yes	1.040	525	50	1,21	0,92-1,61	1,11	0,82-1,49
**Yearly contact with the GP**							
No	55	26	47	1		1	
Yes	1.226	609	50	1,21	0,92-1,61	1,07	0,60-1,89
**Access to the MCHS in the last 5 years**							
No	692	306	44	1		1	
Yes	589	329	56	1,60	1,28-1.99	1,55	1,23-1,95
**Leisure-time physical activity** (2 - ≥7 hours /week)							
No	749	359	48	1		1	
Yes	532	276	52	1,17	0,94-1,46	1,11	0,88-1,41

The response rate to the survey aimed at health care professionals was unsatisfactory, so the results are not representative and are therefore not presented.

### Prescription analysis

**Figure 2:**
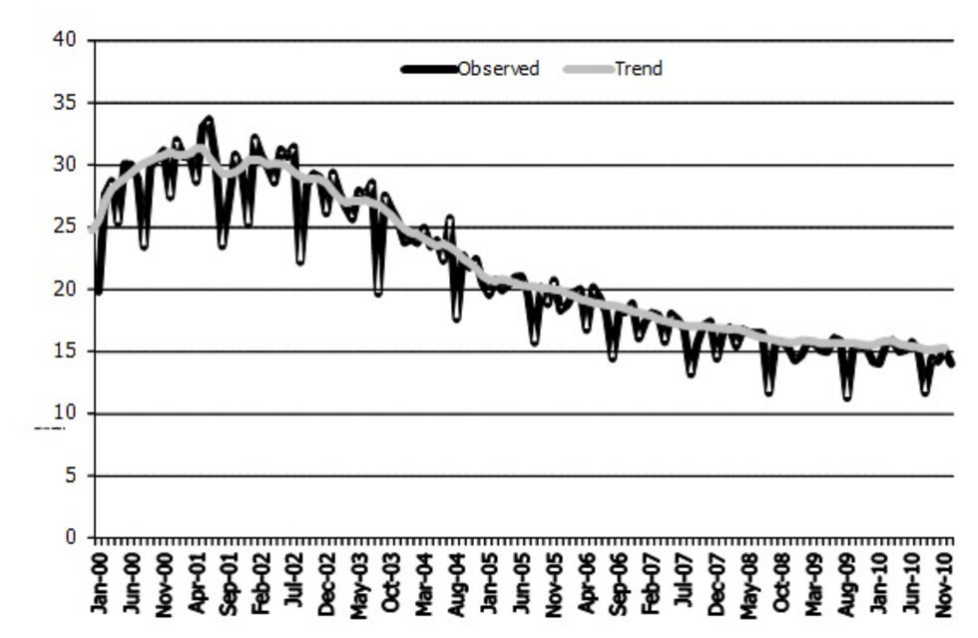
HT prescriptions trend in Italy, years 2000-2010. Legend: Y axis reports DDD/1000 population per day.

HT prescriptions dropped by half, from 30 DDD per 1,000 population per day in 2000-2002, to 15 DDD in 2010, in Italy. This trend was more evident after the publication of the Women’s Health Initiative results, in 2002 (Figure 2). HT exposure in the women aged 45-60 years was estimated from prescription data collected in the eight LHUs in 2006-2010. The prevalence of HT use fell from 6% to 4% in these five years, indicating that approximately 300,000 women were exposed to HT in Italy in 2010. 

The time-series analysis brought to light no differences in HT time trends before and after the intervention, but these results are difficult to assess because of the short observation period, in fact the intervention period lasted only 11 months and the post-intervention observation period was very short.

### Local LHUs’ activities

Activities implemented in the intervention LHUs are reported in table 3. 

**Table 3:** *Know the menopause*: activities of the intervention LHUs.

	**TRAINING COURSES FOR HEALTH PROFESSIONALS**		**N° OF COPIES OF EDUCATIONAL MATERIALS**				**PUBLIC MEETINGS WITH WOMEN**	**DISSEMINATION BY MEDIA**	
	**Target**	**N° of participants / N° invited (%)**	**Leaflets for women**	**Leaflet for physicians**	**Stickers for pharmacies**			**Articles in local bulletins, newspapers, newsletters, web pages**	
**Bergamo**	GPs	485/676 (72)	76,200	480	246		Meetings in public spaces	YES	
(1,103,190 *inh*)	Pharmacists	579/1050 (55)					Ad hoc information when buying drugs for menopause		
	Gynaecologists	14/80 (17)					Ad hoc counselling		
	Midwives	62/180 (34)					Information during cervical cancer screening		
	Nurses	30/nd					Letter to all target women		
**Siena**	GPs	123/220 (56)	18,400	480	85		Meetings in public spaces	NO	
(263,000 *inh*)	Pharmacists	87/456 (19)					Ad hoc information when buying drugs for menopause		
	Gynaecologists	16/27 (59)							
	Midwives	19/36 (53)							
**Roma H**	GPs	110/434 (25)	38,600	735	100		Meetings in public spaces	YES	
(480,000 *inh*)	Pharmacists	17/118 (14)					Ad hoc information when buying drugs for menopause		
	Gynaecologists	24/57 (42)					Ad hoc counselling		
	Midwives	57/67 (85)							
**Enna**	GPs	47/152 (31)	13,300	300	65		Meetings in public spaces	YES	
(180,000 *inh*)	Pharmacists	48/63 (78)					Ad hoc information when buying drugs for menopause		
	Gynaecologists	20/28 (71)					Ad hoc counselling		
	Midwives	19/27 (70)							
**TOTAL**	GPs	765/1482 (52)	146,500	1,995	496				
	Pharmacists	731/1687 (43)							
	Gynaecologists	74/201 (37)							
	Midwives	157/310 (51)							

Inh=inhabitans

## Discussion

This project was designed to disseminate the HT Consensus Conference statements using a multimodal approach, and to assess the effect of this dissemination. It involved the LHUs of the participating regions and, through them, general practitioners, gynaecologists, midwives, and pharmacists. In the intervention LHUs trained health professionals disseminated the CC recommendations to resident women aged 45-64 years. 

The effect of the project activities was evaluated through knowledge, attitude and behavior surveys towards HT and menopause, and through HT prescriptions and feedback from health professionals involved in the dissemination. 

A huge amount of information was spread through the cascade process that reached health professionals, general practitioners, gynaecologists, pharmacists, midwives and women. About 3,700 health professionals were contacted, and about 1,800 participated actively in the project. Printed leaflet on menopause (146,500 copies) was also a key point of the project to convey independent information, and to facilitate the discussion about menopause among women and health care professionals. 

Menopause and HT continued to be an interesting issue for the press but the survey highlighted some limits: clinical experts are still the main source of information, HT is often presented as a drug for prevention, and HT risks are not often mentioned.

The women’s survey highlighted the need for more and better-quality information about menopause, HT and alternative treatments. The lack of information may restrict aware health choices especially for less-educated women that resulted at higher risk of not receiving information both on menopause as well as on hormonal and alternative therapies (data not presented). Interviewed women attended regularly GP praxis, most of them joined female cancer prevention programmes and many underwent a gynaecologic check in the last five years. Therefore, health professionals seem to have several opportunities to proactively provide them information on menopause and HT. Interventions should be made in order to strengthen the advisory role of clinicians, when taking care of women using or considering to use HT because, there is still a need to disseminate evidence-based, clear, accessible information. As in the 2007 survey [12], the women interviewed complained of the persistence of weak and sometimes contradictory information about menopause and HT. 

Over the last decade HT prescriptions decreased mainly because of the publication of the WHI study in which HT use was associated with increases in cardiovascular and cancer risks. Our study was conducted in a period of low HT consumption, so it was difficult to detect differences due to the interventions. Nevertheless, the accessibility of local data will enable us to replicate this model in other geographical area or other pharmaceutical categories.

This study, carried out with limited public resources, has some limitations. The most important limit of the study is the time frame for the project activities. For instance, it was too short to evaluate the impact on drug prescriptions among intervention and control LHUs. Secondly, another limit was the local organizational difficulties arising from the innovative offer of local activities, as well as the paucity of resources dedicated to the project. None of the intervention local units organized to develop educational interventions in schools, because of resource limitations. Finally, the very low response rate to the survey of physicians limited data analysis. Due to organizational difficulties - the medical practitioners’ lists of some LHUs were not up-to-date, in the units it was hard to find people available to coordinate and spread the survey, and interest in this topic was scant, especially among gynaecologists. The limited response might also be tied to the way the questionnaire was administered (by mail or online): face-to-face interviews might give a better response rate.

Even with the limits described, the project showed the feasibility of this kind of intervention within the NHS and offered an opportunity to launch and sustain a multidisciplinary, multimodal approach to menopause. It directly involved those who deal with menopausal women in everyday practice, such as specialists and clinicians, health professionals working in the territorial women’s health centers, as well as women interested in the topic. The framework and the strategy of *Know the menopause* project make it a reference for further studies of health information dissemination.

Finally, in the framework of the project, the issue of menopause was used by health professionals as a means to discuss women’s wellbeing and lifestyle rather than just to assess whether to prescribe a drug. At the end of the project one general practitioner commented “… at the beginning we asked ourselves what point would there be in talking to women about menopause and hormones once again, considering so few women use them in Italy. But soon we realized that this was a unique occasion to talk about menopause in a proper way, considering all the aspects related to this period, informing women about healthy lifestyles”.
